# Calcium balance in peritoneal dialysis patients treated by continuous ambulatory peritoneal dialysis (CAPD) and automated peritoneal dialysis (APD) cyclers

**DOI:** 10.1007/s40620-023-01575-2

**Published:** 2023-03-02

**Authors:** Andrew Davenport

**Affiliations:** 1 UCL Department of Renal Medicine, Royal Free Hospital, University College London Medical School, London, UK; 2 UCL Department of Nephrology, Royal Free Hospital, University College London, Rowland Hill Street, London, NW3 2PF UK

**Keywords:** Peritoneal dialysis, DXA, Calcium, Osteoporosis, Gender, Phosphate binders, Urinary calcium, Ultrafiltration

## Abstract

**Introduction:**

Although vascular calcification is a recognised complication for haemodialysis patients, peritoneal dialysis (PD) patients are also at risk. As such we wished to review peritoneal and urinary calcium balance and the effect of calcium containing phosphate binders (CCPBs).

**Methods:**

Twenty-four-hour peritoneal calcium balance and urinary calcium were reviewed in PD patients undergoing their first assessment of peritoneal membrane function.

**Results:**

Results from 183 patients, 56.3% male, 30.1% diabetic, mean age 59.4 ± 16.4 years, median 2.0 (2–6) months of PD, 29% treated by automated PD (APD), 26.8% continuous ambulatory (CAPD) and 44.2% APD with a day-time exchange (CCPD) were reviewed. Peritoneal calcium balance was positive in 42.6%, and remained positive in 21.3% after including urinary calcium losses. PD calcium balance was negatively associated with ultrafiltration (odds ratio 0.99 (95% confidence limits 0.98–0.99), *p* = 0.005. PD calcium balance was lowest with APD (APD − 0.45 (− 0.78 to 0.05) vs CAPD − 0.14 (− 1.18 to 0.59) vs CCPD − 0.03) − 0.48 to 0.5) mmol/day), *p* < 0.05, with 82.1% of patients with a positive balance prescribed icodextrin, when combining peritoneal and urinary losses. When considering CCPB prescription, then 97.8% of subjects prescribed CCPD had an over-all positive calcium balance.

**Discussion:**

Over 40% of PD patients had a positive peritoneal calcium balance. Elemental calcium intake from CCPB had a major effect on calcium balance, as median combined peritoneal and urinary calcium losses were < 0.7 mmol/day (26 mg), so caution is required to prevent excessive CCPB prescribing, increasing the exchangeable calcium pool and thus potentially increasing vascular calcification, particularly for anuric patients.

**Graphical abstract:**

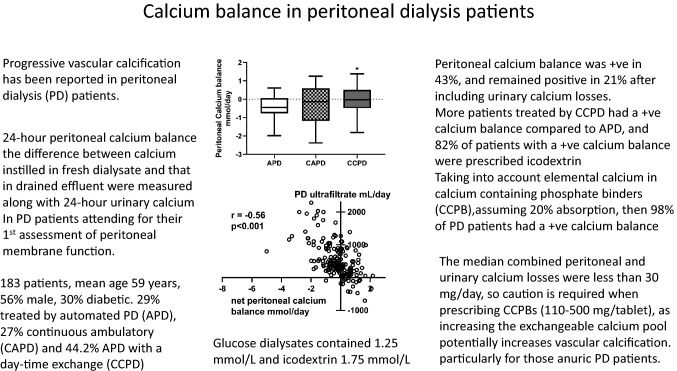

## Introduction

Patients with chronic kidney disease (CKD) are at increased risk of vascular and cardiac valvular calcification. Although initial reports suggested that this risk was greatest for end-stage CKD patients treated by haemodialysis, more recent studies have also shown that peritoneal dialysis (PD) patients are also at increased risk [1]. Historically, PD fluids were formulated with a high dialysate calcium concentration (1.75 mmol/L), as these were developed prior to the introduction of activated forms of vitamin D3 into clinical practice. Due to concerns about a positive calcium gain from these dialysates, lower calcium containing dialysates (1.25 mmol/L) were introduced in the 1990s. However, use of these dialysates was reported to induce a negative calcium balance resulting in increasing parathyroid hormone concentrations [2], and leading to some centres opting to use combinations of both higher and lower calcium dialysates [3].

Although higher calcium dialysates were reported to induce a positive peritoneal calcium balance, and lower calcium dialysates a negative balance, this was not a universal finding, and other factors including serum calcium, PD dwell time, ultrafiltration volumes, and PD modality were also reported to affect peritoneal calcium balance, along with the use of calcium and non-calcium containing phosphate binders [4–6].

The demographics of CKD patients treated by PD has changed over recent times in Europe and North America, with increasing numbers of elderly patients now being treated by PD, with a corresponding increase in the prevalence of both sarcopenia and osteoporosis [7, 8]. As such we wished to revisit calcium balance in a contemporary cohort of PD patients and to determine the potential effect of additional calcium containing phosphate binders.

## Methods

We reviewed the PD calcium balance in a cohort of adult PD patients attending a United Kingdom (UK) university hospital for their first assessment of peritoneal membrane function. All patients had started PD electively, and in addition had undergone dual-energy X-ray absorptiometry (DXA) to assess bone mineral density, as recommended by the Kidney Disease Improving Global Outcomes (KDIGO) CKD-MBD group [10]. The DXA scans were performed after drainage of PD dialysate, post voiding and with patients weighed wearing only a thin gown and height measured using a stadiometer (Hologic Discovery A (S/N87402.1), software version 13.5.2.1, Hologic, USA) [9]. Bone mineral density was measured at the lumbar spine (L1–L5) and femoral neck, and additionally reported as T-scores, the bone density comparison to that of a 30-year-old healthy gender-matched person, and Z scores comparing bone density to the average values for a person of the same age and gender. According to WHO criteria, patients were categorized into three groups: normal bone mineral density with a T-score no less than − 1.0, osteopenia with a T-score between − 1.0 and − 2.5, and osteoporosis for patients with a T-score less than − 2.5, or a Z score of < − 2.0 [10–12]. Appendicular lean mass and body fat were also measured by DXA.

PD adequacy was calculated by standard methods from 24-h urinary collections and corresponding spent PD dialysate samples [13], along with estimated protein nitrogen appearance (PNA) calculated from standard equations [14, 15]. Peritoneal membrane transport was calculated from 4-h peritoneal dialysate dwell and plasma creatine concentrations using a standard 2.0 L 22.7 g/L peritoneal dialysate [13, 14]. Calcium was measured photometrically in serum, peritoneal dialysate and urine (5-nitro-5′-methyl-BAPTA method) (Roche Modular P^®^ analyser, Roche Diagnostics Limited, Burgess Hill, UK), in a UK accredited laboratory. Peritoneal calcium removal was calculated by the difference between the daily amount of calcium instilled in fresh dialysate and the calcium measured in the 24-h effluent dialysate. Patients and staff were instructed to allow 15 s for the flush before fill, continuous ambulatory peritoneal dialysis (CAPD) technique, and the median volume measured was 90 mL, as such calcium balance in CAPD patients was then adjusted from an initial volume of 2.15 L in a fresh dialysate bag [16]. Volumetric measurements were obtained for patients dialysing with automated peritoneal dialysis (APD) cyclers without and with an additional day fill (CCPD). Peritoneal dialysis prescriptions used standard glucose dialysates (calcium 1.25 mmol/L) and icodextrin (1.75 mmol/L) (Baxter Health Care, Deerfield, Illinois, USA). No patient had been treated for PD peritonitis or had an acute hospital admission within the preceding 2 months.

Hospital computerised records were reviewed to retrieve patient demographics, relevant laboratory investigations and medications. Daily ingestion from medications was estimated from the elemental calcium content of prescribed calcium containing phosphate binders (calcium carbonate, calcium acetate and combination of calcium and magnesium carbonate). Dietary absorption of elemental calcium has been estimated between 20 and 40%, but is decreased in patients with CKD due to Vitamin D deficiency and to prevent calcium overload [17]. However, studies have shown that although absorption of normal amounts of dietary calcium are reduced, when large doses of calcium are administered, as with calcium containing phosphate binders, then absorption is similar to healthy individuals [18]. As such we have estimated elemental calcium absorption at 20%. Patient co-morbidity was assessed by Stoke-Davies and patient functionality by the Clinical Frailty Scale scores [19, 20].

### Statistical analysis

Normally distributed continuous variables were expressed by mean values ± standard deviation (SD), and non-parametric continuous variables reported as median (25th and 75th percentile). Categorical variables were expressed by frequencies and percentages. Standard analyses included *t*-test, and ANOVA for parametric continuous variables, Mann–Whitney *U* test and Kruskal–Wallis for nonparametric continuous variables, and the chi-square (*X*^2^) test was performed for categorical variables. Tukey and Games-Howell adjustments were made in cases of multiple testing. Univariate analysis was carried out by Spearman correlation. Determinants of a positive peritoneal and urinary calcium balance, and then overall calcium balance considering the elemental calcium content of calcium containing phosphate binders were analysed using a step backward multivariable logistic regression using variables associated with *p* < 0.1 on univariate analysis. Variables were then excluded if not statistically significant, unless they improved model fit. If required, nonparametric variables were log transformed. Analyses were performed using Statistical Package for Social Sciences (SPSS Version 28.0 software, IBM Corp., Armonk, New York, USA), Prism 9.4 (Graph Pad, San Diego, USA) and Microsoft Excel Version 2107 (Build 14,228.20226). A two-tailed *p* value < 0.05 was considered statistically significant.

### Ethics

This retrospective audit was conducted according to United Kingdom (UK) National Research Ethics guidelines and did not require additional local ethical approvals or individual patient consent. The audit was registered with the University hospital, and all patient data was anonymised in keeping with UK regulations for audit and service development.

## Results

**Fig. 1:**
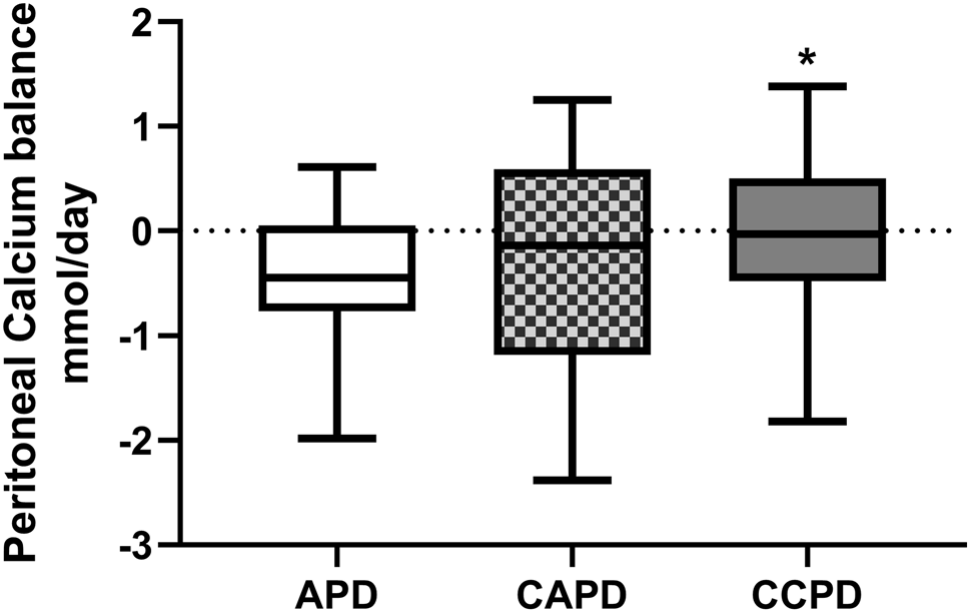
Net peritoneal calcium balance according to peritoneal dialysis (PD) modality; Net balance is the difference in the amount of calcium instilled in the fresh dialysate and that drained in 24 h. Automated PD with a dry day (APD), continuous ambulatory PD (CAPD), automated PD with a daytime exchange (CCPD). APD patients all used lower calcium dialysates (1.25 mmol/L) and the majority had a negative balance, whereas nearly all CAPD and CCPD patients used a long icodextrin exchange (1.75 mmol/L), and fewer patients treated by CAPD and CCPD had a negative daily peritoneal calcium balance. Median, interquartile and 95% confidence limits. *p < 0.05 vs APD

We reviewed the results from 183 adult PD patients who underwent their first assessment of peritoneal membrane function between July 2016 and April 2021, median 3 (2–7) months after starting PD, who had a DXA scan 2 (2–4) months after starting PD (Table 1). Seventy-eight (42.6%) patients had a daily positive peritoneal calcium balance, 19 of 53 (35.8%) APD, 20 of 48 (41.7%) CAPD and 39 of 82 (47.6%) CCPD (Fig. 1). When urinary calcium losses were considered, then 39 (21.3%) had a daily combined peritoneal and urinary positive calcium balance (Table 1). Patients with a positive calcium balance had lower urine volumes and urinary calcium and peritoneal ultrafiltration and calcium removal. Fewer patients were treated by APD, and more used icodextrin, a higher calcium dialysate. These patients had lower serum calcium levels and lower LS and FN DXA Z scores, and more patients had been prescribed calcium containing phosphate binders. Body weight was not significantly lower, and although PNA was lower, when adjusted for body weight (nPNA) it did not differ (0.90 ± 0.24 vs 0.94 ± 0.24 g/kg/day).

**Table 1:** Patient demographics and body composition and bone densitometry measured by dual-energy x-ray absorptiometry (DXA), with patients divided into those according to net calcium peritoneal and urinary balance

Variable	All patients	− ve Ca balance	+ ve Ca balance
Number (%)	183	144 (78.7)	39 (21.3)
Male (%)	103 (56.3)	78 (54.2)	25 (64.1)
Diabetic (%)	55 (30.1)	46 (31.9)	9 (23.1)
White/Black/Asian %	47/29.5/23.5	49.3/28.5/22.2	38.5/33.3/28.2
Age years	59.4 ± 16.4	60.6 ± 16.0	54.9 ± 18.0
PD treatment months	2.0 (2.0–6.0)	6.0 (2–29.5)	9 (3–20)
Urine volume mL/day	1147 (568–1718)	1215 (669–1741)	96 (476–1553)*
Kt/Vurea urine	1.3 (0.8–2.02)	1.46 (0.85–2.06)	0.96 (0.61–1.73)
Kt/Vurea peritoneal	1.1 (0.83–1.41)	1.08 (0.87–1.35)	1.23 (0.79–1.55)
Kt/Vurea total	2.37 (1.89–1.41)	2.45(1.95–3.08)	2.16 (1.84–2.73)
PNA g/day	65.6 ± 19.1	67.3 ± 19.2	59.3 ± 17.9**
4 h D/Pcreatinine	0.72 ± 0.14	0.72 ± 0.14	0.73 ± .15
APD/CAPD/CCPD (%)	29/26.8/44.2	33.3/27.1/38.6	12.8/25.6/61.6*
PD dialysate usage L/day	9.2 (7.3–11.10	9.1 (7.3–11.0)	9.7 (7.7–12.1)
Icodextrin usage (%)	119 (65)	87 (60.4)	32 (82.1)**
22.7 g/L dextrose usage	51 (27.9)	37 (25.7)	14 (35.9)
24 h PD ultrafiltrate mL	395 (200–817)	400 (240–883)	300 (62–700)*
Weight kg	70.8 ± 14.5	72.4 ± 16.2	66.7 ± 12.7
Body fat kg	20.1 (18.8–25.5)	21.5(15.6–27.2)	16.1(9.8–25.3)
ALM kg/	19.3 ± 4.9	19.7 ± 5.1	17.7 ± 3.9
C reactive protein mg/L	4 (1–9)	3.0 (1.0–5.0)	3.0 (1.0–8.0)
PTH pmol/L	27.1 (16.1–40.2)	27.5 (16.3–42.2)	26.7 (15.7–37.1)
Creatinine umol/L	580 (453–787)	565 (453–767)	616 (458–911)
Glucose mmol/L	5.7 (4.7–7.4)	5.8 (4.7–7.5)	5.6 (4.7–6.4)
LS BMD g/cm^2^	1.03 ± 0.20	1.04 ± 0.21	0.98 ± 0.17
FN BMD g/cm^2^	0.74 ± 0.14	0.75 ± 0.15	0.71 ± 0.11
T score LS	− 0.7 (− 1.8 to 0.4)	− 0.7 (− 1.6 to 0.5)	− 1.1 (− 2.0 to 0)
T score FN	− 1.4 (− 2.1 to − 0.8)	− 1.3(− 2.1 to − 0.8)	− 1.6 (− 2.1 to − 1.0)
Z score LS	0.1 (− 1.0 to 1.3)	0.3 (− 0.9 to 0.3)	− 0.7(− 1.3 to 0.6)*
Z score FN	− 0.5(− 0.9 to 0.2)	− 0.3 (− 0.9 to 0.3)	− 0.7(− 1.1 to − 0.3)*
Haemoglobin g/L	110.0 ± 15.9	110.9 ± 15.2	111.0 ± 13.6
Albumin g/L	37.3 ± 4.3	37.5 ± 4.2	36.4 ± 4.3
Calcium mmol/L	2.30 ± 0.18	2.33 ± 0.17	2.17 ± 0.13***
Phosphate mmol/L	1.52 ± 0.41	1.53 ± 0.42	1.47 ± 0.41
Magnesium mmol/L	0.85 ± 0.15	0.86 ± 0.15	0.84 ± 0.16
Bicarbonate mmol/L	25.4 ± 3.2	25.0 ± 3.0	26.0 ± 3.0
PTH pg/mL	27.1(11.6–40.2)	27.5 (16.3–42.2)	26.7 (15.7–37.1)
Elemental calcium g/day	0.77 ± 1.11	0.41 ± 0.66	1.59 ± 1.28
Alfacalcidol ug/week	1.0 (0–3.0)	0.75 (0–3.0)	1.75 (0− 3.0)
Cholecalciferol ug/day	71.4(0−71.4)	71.4 (0–71.4)	71.4 (0–71.4)
Calcium binders prescribed (%)	90 (49.2)	58 (40.3)	32 (82.1)**
Davies co-morbidity	1.0 (0–2.0)	1.0 (0–2.0)	1.0 (0–2.0)
Frailty score	3.0 (2.0–4.0)	3.0 (2.0–4.0)	3.0 (2.0–4.0)
Urine calcium mmol/day	0.49 (0.1–0.9)	0.6(0.2–1.1)	0.1(0.1–0.4)***
Net peritoneal Calcium balance mmol/day	− 0.15 (− 0.8 to 0.4)	− 0.4(− 1.0 to 0.1)	0.7(0.4–1.2)***

Data expressed as integers, percentages, mean ± standard deviation and median (interquartile range). T and Z scores according to world health organization definitions [9]. Comparison vs − ve calcium balance *p < 0.05, **p < 0.01, ***p < 0.001

*PD* peritoneal dialysis, *Kt/V* weekly urea clearance, *4hrD/Pcreatinine* peritoneal equilibration test, *PNA* protein nitrogen appearance rate, *APD* automated PD with a dry day, *CAPD* continuous ambulatory PD, *CCPD* automated PD with a daytime exchange, *ALM* appendicular lean mass, *CRP* C reactive protein, *PTH* parathyroid hormone, *alphacalcidol* activated vitamin D3, *elemental calcium* elemental calcium in medications, *LS* lumbar spine, *FN* femoral neck, *BMD* bone mineral density

Elemental calcium from calcium-based phosphate binders was calculated, and assuming that only 20% of this calcium could be absorbed, the number of patients with a positive calcium balance considering peritoneal and urinary calcium fluxes and elemental calcium intake increased to 105 (57.4%) (Table 2). Only 2 (2.2%) patients who were prescribed calcium containing phosphate binders had a net calcium loss, when considering calcium absorption from the phosphate binders. Patients with a positive calcium balance had lower peritoneal and urinary calcium losses, lower serum calcium and urinary urea and total urea clearance, lower LS Z scores, body fat, and fewer were diabetic (Table 2). Besides greater prescription of calcium-based phosphate binders and alfacalcidol, and elemental calcium intake, patients had greater peritoneal urea clearance, higher haemoglobin and serum creatinine.

**Table 2:** Patient demographics and body composition and bone densitometry measured by dual-energy x-ray absorptiometry (DXA), with patients divided into those according to net calcium peritoneal and urinary balance and prescribed elemental calcium from calcium containing phosphate binders

variable	All patients	− ve Ca balance	+ ve Ca balance
Number (%)	183	78 (63.9)	105 (36.1)
Male (%)	103 (56.3)	38 (48.7)	64 (61.0)
Diabetic (%)	55 (30.1)	30 (38.5)	25 (23.8)*
White/Black/Asian %	47/29.5/23.5	52.6/25.6/21.8	42.9/32.3/24.8
Age years	59.4 ± 16.4	61.6 ± 16.2	57.8 ± 16.4
PD treatment months	2.0 (2.0–6.0)	2.0 (2.0–4.0)	2.0 (2.0–7.0)
Urine volume mL/day	1147 (568–1718)	1183 (762–1762)	1088 (508–1659)
Kt/Vurea urine	1.3 (0.8–2.02)	1.60 (0.90–2.2)	1.12 (0.69–1.83)*
Kt/Vurea peritoneal	1.1 (0.83–1.41)	1.03 (0.78–1.31)	1.21 (0.90–1.48)*
Kt/Vurea total	2.37 (1.89–1.41)	2.62 (2.10–3.10)	2.23 (1.84–2.92)*
PNA g/day	65.6 ± 19.1	69.4 ± 20.2	63.5 ± 18.1
4 h D/Pcreatinine	0.72 ± 0.14	0.71 ± 0.14	0.72 ± 0.14
APD/CAPD/CCPD (%)	29/26.8/44.2	35.9/28.2/35.9	23.8/24.7/51.5
PD dialysate usage L/day	9.2 (7.3–11.10)	9.0 (7.2–10.9)	9.6 (8.0–11.5)
Icodextrin usage (%)	119 (65)	45 (57.7)	74 (70.5)
22.7 g/L dextrose usage	51 (27.9)	17 (21.8)	34 (32.4)
24 h PD ultrafiltrate mL	395 (200–817)	400 (243–639)	380 (138–830)
Weight kg	70.8 ± 14.5	72.5 ± 15.4	69.7 ± 14.0
Body fat kg	20.1 (18.8–25.5)	22.3 (17.6–26.4)	18.5 (13.2–23.7)**
ALM kg/	19.3 ± 4.9	18.9 ± 4.9	19.6 ± 5.0
C reactive protein mg/L	4.0 (1.0–9.0)	3.0 (1.0–6.0)	3.0 (1.0–8.0)
PTH pmol/L	27.1 (16.1–40.2)	26.7 (17.1–43.2)	27.6 (15.0–38.9)
Creatinine umol/L	580 (453–787)	520 (394–648)	683 (484–919)***
Glucose mmol/L	5.7 (4.7–7.4)	6.1 (4.9–8.0)	5.6 (4.7–6.9)
LS BMD g/cm2	1.03 ± 0.20	1.05 ± 0.22	1.01 ± 0.19
FN BMD g/cm2	0.74 ± 0.14	0.74 ± 0.16	0.74 ± 0.13
T score LS	− 0.7 (− 1.8 to 0.4)	− 0.7 (− 1.5 to 0.5)	− 0.7 (− 1.9 to 0.3)
T score FN	− 1.4 (− 2.1 to − 0.8)	− 1.3 (− 2.1 to − 0.8)	− 1.4(− 2.0 to − 0.8)
Z score LS	0.1 (− 1.0 to 1.3)	0.4 (− 0.8 to 1.7)	− 0.3(− 1.3 to 1.1)*
Z score FN	− 0.5 (− 0.9 to 0.2)	− 0.3 (− 0.9 to 0.3)	− 0.5 (− 1.0 to 0.2)
Haemoglobin g/L	110.0 ± 15.9	108.0 ± 14.0	113.0 ± 15.0*
Albumin g/L	37.3 ± 4.3	37.8 ± 4.2	36.9 ± 4.3
Calcium mmol/L	2.30 ± 0.18	2.37 ± 0.16	2.25 ± 0.17***
Phosphate mmol/L	1.52 ± 0.41	1.47 ± 0.44	1.55 ± 0.39
Magnesium mmol/L	0.85 ± 0.15	0.84 ± 0.13	0.86 ± 0.16
Bicarbonate mmol/L	25.4 ± 3.2	25.0 ± 3.3	26.1 ± 3.0*
PTH pg/mL	27.1 (11.6–40.2)	26.7 (17.1–43.2)	27.6 (15.0–38.9)
Elemental calcium g/day	0.77 ± 1.11	0.41 ± 0.66	1.59 ± 1.28***
Alfacalcidol ug/day	0.15 (0–0.43)	0.1 (0–0.25)	0.25 (0.04–0.5)**
Cholecalciferol ug/day	71.4(0–71.4)	71.4 (0–71.4)	71.7 (0–71.4)
Calcium binders prescribed (%)	90 (49.2)	2 (2.6)	88 (83.8)***
Davies co-morbidity	1.0 (0–2.0)	1.0 (0−2.0)	1.0 (0–2.0)
Frailty score	3.0 (2.0–4.0)	3.0 (2.0–4.0)	3.0 (2.0–4.0)
Calcium urine mmol/day	0.49 (0.1–0.9)	0.6 (0.3–1.2)	0.3 (0.1–0.9)*
Net peritoneal Calcium balance mmol/day	− 0.15 (− 0.8 to 0.4)	− 1.1 (− 2.1 to 0.6)	− 0.5 (− 1.4 to 0.2)***

Data expressed as integers, percentages, mean ± standard deviation and median (interquartile range). T and Z scores according to world health organization definitions [9]. Comparison vs − ve calcium balance *p < 0.05, **p < 0.01, ***p < 0.001

*PD* peritoneal dialysis, *Kt/V* weekly urea clearance, *4hrD/Pcreatinine* peritoneal equilibration test, *PNA* protein nitrogen appearance rate, *APD* automated PD with a dry day, *CAPD* continuous ambulatory PD, *CCPD* automated PD with a daytime exchange, *ALM* appendicular lean mass, *CRP* C reactive protein, *PTH* parathyroid hormone, *alphacalcidol* activated vitamin D3, *elemental calcium* elemental calcium in medications, *LS* lumbar spine, *FN* femoral neck, *BMD* bone mineral density

On univariate analysis, daily combined peritoneal and urinary calcium balance was associated with a positive peritoneal calcium balance and icodextrin usage, and negatively with body composition, particularly lean mass, urinary volume and calcium, PNA, FN T and Z scores, serum calcium and phosphate and age (Table 3). When the estimated amount of elemental calcium absorbed from calcium containing phosphate binders was added to the overall daily calcium balance, this had a major effect (Table 3).

**Table 3:** Spearman rho univariate association with positive calcium balance (net peritoneal and urinary) and then including elemental calcium intake in calcium containing phosphate binders

Variables	Positive calcium balance Peritoneal and urine		Positive calcium balance Including calcium binders	
	rho	*p*	rho	*p*
PD Calcium balance mmol/day	0.76	< 0.001	0.35	< 0.001
Urinary calcium mmol/day	− 0.48	< 0.001	− 0.19	0.011
Serum calcium mmol/L	− 0.43	< 0.001	− 0.32	< 0.001
Femoral Z score	− 0.32	< 0.001	− 0.14	0.086
Left arm lean mass kg	− 0.29	0.001	− 0.10	0.26
PD Ultrafiltration mL/day	− 0.27	0.003	− 0.07	0.35
R arm lean mass kg	− 0.27	0.003	− 0.13	0.16
Lean weight kg	− 0.25	0.003	0.07	0.43
Urine mL/day	− 0.22	0.004	− 0.15	0.044
Appendicular lean mass kg	− 0.21	0.017	0.07	0.34
PNA g/day	− 0.20	0.008	− 0.07	0.02
Serum phosphate mmol/day	− 0.17	0.026	0.07	0.34
Femoral T score	− 0.16	0.045	− 0.07	0.39
Age years	− 0.15	0.037	− 0.12	0.11
Icodextrin usage L/day	0.15	0.043	− 0.15	0.034
Elemental calcium mg/day	0.07	0.38	0.91	< 0.001
Serum creatinine umol/L	0.08	0.31	0.31	< 0.001

Elemental calcium estimated from prescribed calcium containing phosphate binders

*PD* peritoneal, *PNA* protein nitrogen accumulation

These variables were then entered into logistic multivariable models to determine which factors were independently associated with a positive calcium balance. Considering urinary and calcium balances, a positive peritoneal and urinary calcium balance was associated with CCPD, and negatively with peritoneal ultrafiltration, serum calcium and PNA (Table 4). When considering a positive calcium balance after including elemental calcium from phosphate binders, the prescription of calcium containing phosphate binders was very strongly associated with a positive balance, whereas 24-h peritoneal calcium loss and urinary volume were associated with a negative balance.

**Table 4:** Logistic multivariable regression analysis of variables independently associated with a positive calcium balance (net peritoneal and urinary) and then including elemental calcium intake in calcium containing phosphate binders

Variable	*β*	StE *β*	Wald	OR	95% CI	*p*
Net peritoneal and urinary balance						
PD ultrafiltration mL/day	− 0.002	.001	7.8	0.99	0.98–0.99	0.005
PNA g/day	− 0.06	0.02	13.5	0.94	0.94–0.98	0.004
Serum calcium mmol/L	− 7.5	2.04	5.9	0.001	0.00–0.03	< 0.001
PD mode (CCPD)	0.84	0.34	13.7	2.3	1.18–4.52	0.015
Peritoneal and urinary and calcium binders						
Calcium binders yes vs no	8.5	1.9	20.9	4999	130–19,200	< 0.001
PD calcium balance mmol/day	3.8	0.99	1.45	44.1	6.3–309	< 0.001
Log urine output mL/day	− 3.6	1.11	10.5	0.03	0.003–0.24	0.001

Model *r*^2^ 0.47 and 0.86 respectively

*PD* peritoneal dialysis, *PNA* protein nitrogen accumulation, *CCPD* automated PD cycler with additional day exchange

## Discussion

Medial vascular calcification was first described in diabetic patients and associated with peripheral vascular disease. Patients with CKD, and those treated by haemodialysis, and PD are also at increased risk of vascular calcification [1]. Longitudinal studies in PD patients have demonstrated an increased prevalence of cardiac valve and arterial calcification with time [21]. Interestingly, the study by Gallieni M et al., did not demonstrate any association between progressive vascular calcification and serum calcium phosphate or PTH concentrations [21]. As peritoneal dialysates contain calcium concentrations ranging between 1.25 AND 1.75 mmol/L, this may be above the normal serum ionised calcium range of 1.2–1.4 mmol/L, thereby potentially allowing calcium to diffuse from the peritoneal dialysate. Historically, glucose-containing PD dialysates had a calcium concentration of 1.75 mmol/L. Studies involving CAPD patients who were switched from 1.75 to 1.25 mmol/L dialysates [22], or comparing patients using both dialysates [23], showed that although PTH values increased, this could be compensated by increasing alfacalcidol prescription, and no changes in bone biopsy histology were reported [23].

More patients treated by CCPD had a positive peritoneal calcium balance when compared to APD, with all but 4 (5.1%) CCPD patients receiving icodextrin with a concentration of 1.75 mmol/L as the day exchange. Although we found no difference between APD and CAPD, a much smaller study reported greater peritoneal calcium removal with CAPD, however these APD and CAPD patients used the same glucose dialysates [4], whereas the great majority of our CAPD patients (93.8%) used one or more icodextrin exchanges. However, by not taking into account the additional volume in CAPD dialysate bags to allow for the flush before fill technique, this would have led to an overestimation of calcium removal reported by CAPD in this earlier study. In keeping with previous reports, calcium removal by PD was associated with both peritoneal ultrafiltration [4, 5, 19, 20] and higher serum calcium [4, 5]. Previous studies have reported that higher glucose dialysates increase calcium removal, but this was related to greater peritoneal ultrafiltration, particularly with 38.6 g/L dextrose [24, 25].

Although ultrafiltration is an important factor for peritoneal clearance of calcium, we also found that serum calcium concentration was also independently associated with calcium balance. This supports other studies which have reported that peritoneal calcium loss is greater in patients with higher serum calcium concentrations, as a higher serum calcium level would potentially influence diffusive calcium clearance, limiting or even reversing calcium influx from the peritoneal dialysate [4, 5].

Just over half of our patients had a negative peritoneal calcium balance, and this increased to just below 80% when urinary calcium losses were included. So, although urinary losses increased the proportion of patients achieving a net calcium loss with PD, PD losses made a greater contribution to the combination, due to the reduced amount of calcium in the urine of CKD patients [26]. However, the amount of elemental calcium in calcium containing phosphate binders dwarfed peritoneal and urinary losses, with only 2% of patients prescribed calcium containing phosphate binders having a negative calcium balance. Studies in CKD patients have demonstrated the role of 1,25 (OH)_2_ vitamin D3 in increasing intestinal calcium absorption [27]. Although there was no difference in the prescription of alfacalcidol (activated Vitamin D3) in determining peritoneal or urinary calcium losses, when including elemental calcium in prescribed phosphate binders then those patients with a positive balance were prescribed more alfacalcidol. Balance studies in CKD patients have demonstrated that administration of calcium containing phosphate binders leads to a substantially increased positive balance when compared to healthy controls [28, 29]. Our data also show that almost all patients were in a positive calcium balance when prescribed calcium containing phosphate binders. A positive calcium balance will increase the exchangeable calcium pool, thus potentially leading to increased vascular calcification. So, although patients with more osteoporosis, defined by more negative DXA lumbar spine and femoral neck Z scores, had positive peritoneal and urinary calcium balance, an increase in the exchangeable calcium pool does necessarily result in improved bone mineralisation [29, 30]. As this was a cross-sectional study, we cannot comment on whether a positive balance led to an improvement in DXA Z scores. Indeed, many studies have highlighted the relationship between osteoporosis and increased vascular calcification, so simply aiming for a positive calcium balance in PD patients may not reduce the risk of osteoporosis, but potentially increase vascular calcification [31, 32].

This was a cross-sectional study including patients recently starting PD, so most patients had some residual renal function, and as such many patients had some urinary calcium losses to mitigate against a positive peritoneal calcium balance. Although PD ultrafiltration was an important factor in determining peritoneal calcium losses, higher serum calcium also had an effect on increasing peritoneal calcium losses. Peritoneal calcium losses were greater with APD compared to CAPD and CCPD, although whether this was due to differences in dwell times between PD modalities or to the use of icodextrin with a higher dialysate calcium concentration used by CAPD- and CCPD-treated patients remains to be determined. We were unable to collect accurate dietary data to estimate dietary calcium intake and overall calcium balance. The European Best Practice Guideline group recommended that the total intake of elemental calcium should not exceed 2000 mg/day, including calcium obtained from calcium-based phosphate binders [33]. Other studies in dialysis patients have reported that dietary calcium intake was usually below 1000 mg/day, [34], in keeping with UK recommendations of 700 mg/day for adults aged 19–64 years [35]. We were unable to collect detailed information on the dietary calcium intake from our multi-ethnic patient population. However, not all dietary calcium is absorbed, and this may vary not only with age, but can also be lower with higher intakes, (45% with 200 mg/day to 15% with 2000 mg/day). In addition, some food stuffs reduce absorption, such as those containing phytates, and oxalate.

**Fig. 2:**
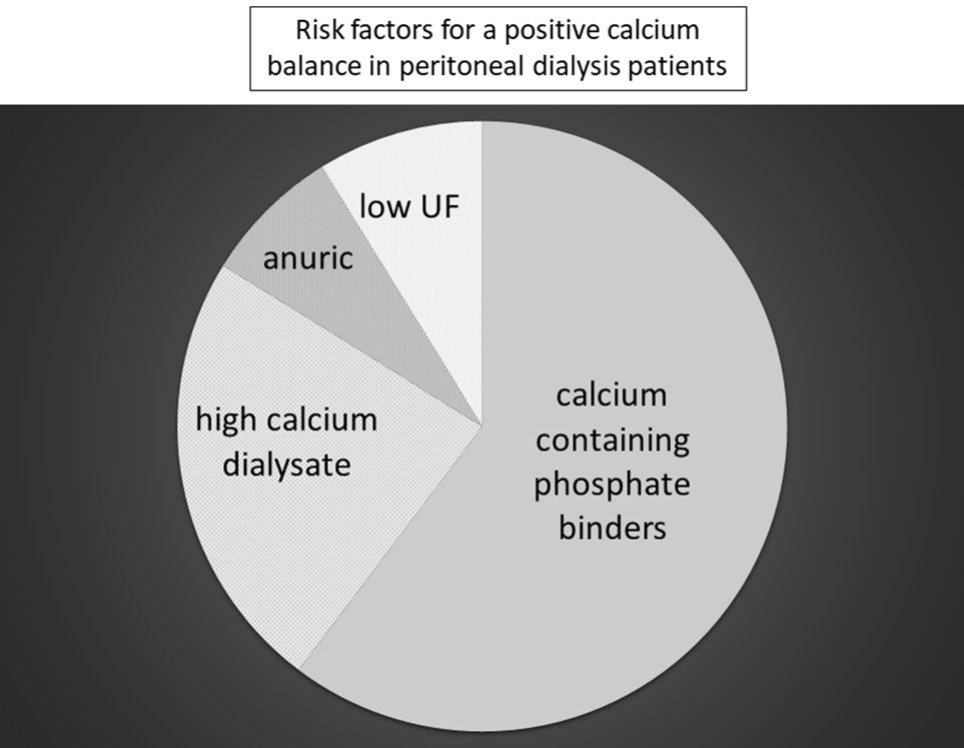
Risk factors for a positive calcium balance in peritoneal dialysis patients

Although vascular calcification is a well recognised complication for haemodialysis patients, PD patients are also equally at risk of progressive vascular and cardiac valvular calcification [21]. Just over 40% of our patients had a positive peritoneal calcium balance. More patients treated by APD, only using 1.25 mmol/L calcium glucose dialysates, had a negative peritoneal calcium balance, whereas fewer patients treated by CCPD using a long day dwell with a 1.75 mmol/L icodextrin dialysate had a negative balance. More than 80% of patients with a positive balance were prescribed icodextrin. Thus, the calcium content of PD dialysates has a major influence on calcium balance. Unless patients have a high daily peritoneal ultrafiltration volume or residual renal function, then the prescription of higher calcium dialysates will result in a positive calcium balance. As the median combined peritoneal and urinary calcium loss was less than 30 mg/day, the prescription of calcium containing phosphate binders, with an elemental calcium content ranging between 110 and 500 mg/tablet, would have risked a positive calcium balance (Fig. 2). As such, more thought is required when prescribing calcium containing phosphate binders to PD patients to prevent excessive calcium loading and increasing the exchangeable calcium pool thereby potentially increasing the risk of progressive vascular calcification.

## Data Availability

Data held UCL Department of Nephrology V drive, data availability upon reasonable request and within NHS guidelines.

## Declarations

### Additional declarations

Retrospective audit complied with UK National Research Ethics guidelines.
